# Finite Impulse Response Filter-Based Track Formation for Preceding Vehicle Tracking Using Automotive Radars

**DOI:** 10.3390/s22020578

**Published:** 2022-01-12

**Authors:** Jung Min Pak

**Affiliations:** Department of Electrical Engineering, Wonkwang University, 460 Iksan-daero, Iksan 54538, Korea; destin11@wku.ac.kr

**Keywords:** automotive radars, finite impulse response (FIR) filter, preceding vehicle tracking, track formation

## Abstract

Automotive radars, which are used for preceding vehicle tracking, have attracted significant attention in recent years. However, the false measurements that occur in cluttered roadways hinders the tracking process in vehicles; thus, it is essential to develop automotive radar systems that are robust against false measurements. This study proposed a novel track formation algorithm to initialize the preceding vehicle tracking in automotive radar systems. The proposed algorithm is based on finite impulse response filtering, and exhibited significantly higher accuracy in highly cluttered environments than a conventional track formation algorithm. The excellent performance of the proposed algorithm was demonstrated using extensive simulations under real conditions.

## 1. Introduction

An automotive radar is a system for detecting obstacles around a host vehicle on the road using microwaves. Currently, automotive radars have attracted increasing attention, and have been applied for tracking the position and velocity of preceding vehicles [1,2,3,4,5,6,7,8]. As many vehicles on the road are equipped with automotive radars, radar signals emitted from the radar of other vehicles may be detected by the receiver of the host vehicle. These signals result in false measurements, which hinder the detection and tracking of true targets [4,5]. In addition, the scattered reflection of radar signals in cluttered road environments may cause increasing false measurements [7]. Thus, it is essential to develop or improve automotive radar systems that are robust against the false measurements [8].

Received radar signals may or may not include the signals from a true target. Thus, it is essential to develop a process that can determine the presence of a signal from a true target in the received signals, and this process in known as the detection process. Thresholding is a representative and simple detection process, and various signal processing techniques, including artificial intelligence algorithms, have been utilized for the detection process [1,3,9,10]. However, because the detected measurements include false measurements, the number of detected measurements can be larger than the actual number of targets. After the detected measurements are obtained, a tracking process is performed.

Various tracking algorithms have been investigated and developed. For example, the probabilistic data association filter (PDAF) [11] has been developed as a representative algorithm for tracking a single target in a clutter [12]. In addition, joint probabilistic data association [13], multiple hypothesis tracking [14], and probability hypothesis density filter [15] have been developed for tracking multiple targets. Generally, tracking algorithms are based on state estimation (also known as stochastic filtering), which requires an initial state estimate and estimation error covariance. The process of generating the initial estimate using the detected measurements is known as the track formation. However, a large number of false measurements in highly cluttered environments hinders the track formation process [12].

This study proposed a novel track formation algorithm to address the problem of tracking a single preceding vehicle using automotive radars. Currently, two representative conventional algorithms are available for this problem [12]: The cascaded logic track formation, which is a heuristic algorithm, and the multiple model track formation (MMTF), which is an advanced algorithm based on the interacting multiple model (IMM) method [12]. These methods utilize the two-point differencing method, which computes the initial estimate of the velocity by differencing two successive measurements. In contrast, the algorithm proposed in this study is based on finite impulse response (FIR) filtering, which has recently attracted attention from researchers [16,17,18,19,20,21,22,23,24]. FIR filters are bach-processing filters, which can produce state estimate and error covariance using recent finite measurements, without filter initialization. Inspired by these characteristics of the FIR filter, in this study, we designed a novel FIR filter-based track formation (FFTF) algorithm. The FFTF algorithm proposed in this study exhibited a significantly higher accuracy than the conventional MMTF under highly heavy clutter conditions. Moreover, despite changes in the relative distance and velocities between vehicles, the FFTF provided consistent track formation. The excellent performance of the FFTF was demonstrated using extensive simulations that considered various real-world conditions.

This paper is organized as follows. Section 2 introduces the track formation problem for a single preceding vehicle using automotive radars in cluttered environments. Section 3 describes the MMTF, and Section 4 proposes the FFTF algorithm. Section 5 presents simulation results to demonstrate the performance of the FFTF, and the conclusions are state in Section 6.

## 2. Preceding Vehicle Tracking Using Automotive Radars in Clutters

**Figure 1:**
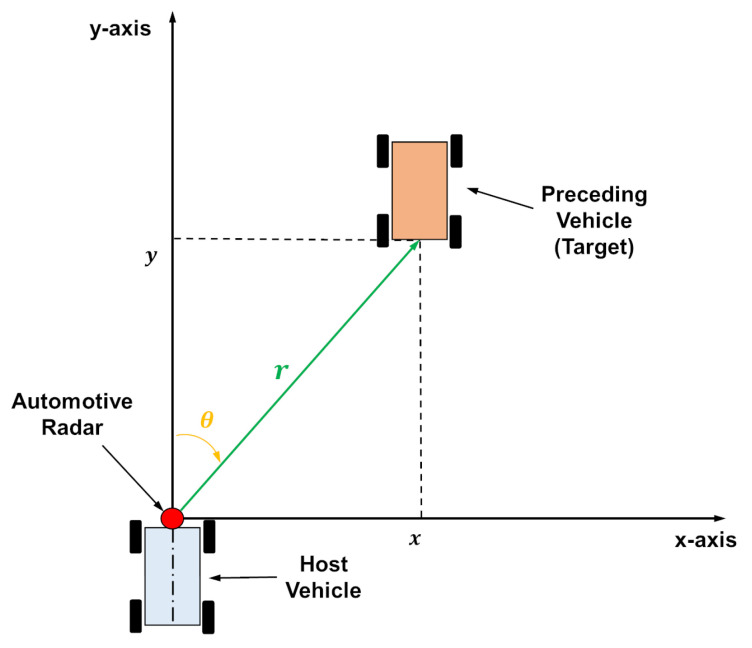
Automotive radar geometry.

Generally, an automotive radar for preceding vehicle tracking is installed in the middle front of the host vehicle, as shown in Figure 1 [20]. The automotive radar emits millimeter waves with a certain field of view (FOV), after which the radar waves scatter and are reflected from various objects on a road. The reflected signals from nontarget objects are referred to as clutters, which hinder the tracking of preceding vehicle. Moreover, owing to the widespread use of automotive radars, the emission of signals from the radars of other vehicles results in the formation of increased clutters. Therefore, this study aimed to estimate the position and velocity of a preceding target vehicle in highly-heavy clutter environment. The position of the preceding vehicle is represented in a two-dimensional (2D) Cartesian coordinate system, as shown in Figure 1. The origin was located on the position of the radar, and the x-axis was aligned with the longitudinal direction of the host vehicle. The radar measured the range, *r*, and bearing, θ, between the host and preceding vehicles, and the measurements included noise. The measured range and bearing, $r_{m}$ and $\theta_{m}$, can be represented as

(1) $$\begin{array}{cc} r_{m} & =r+{\bar{v}}_{r},\\ \theta_{m} & =\theta +{\bar{v}}_{\theta }, \end{array}$$

where ${\bar{v}}_{r}$ and ${\bar{v}}_{\theta }$ are the measurement noises, which are assumed to be the independent and zero mean white Gaussian noises with covariances $\sigma_{r}^{2}$ and $\sigma_{\theta }^{2}$, respectively, [12].

To obtain the preceding vehicle positions in the Cartesian coordinate, the range and bearing measurements were converted as follows:

(2) $$\begin{array}{cc} x_{m} & =r_{m}\;cos\left(\theta_{m}\right),\\ y_{m} & =r_{m}\;sin\left(\theta_{m}\right), \end{array}$$

where $x_{m}$ and $y_{m}$ are the converted measurements, and they can be also represented as:

(3) $$\begin{array}{cc} x_{m} & =x+{\bar{v}}_{x},\\ y_{m} & =y+{\bar{v}}_{y}, \end{array}$$

where ${\bar{v}}_{x}$ and ${\bar{v}}_{y}$ are the measurement noises in the Cartesian coordinate. The measurement vector was defined as $z={\left[x_{m}\;y_{m}\right]}^{T}$, and the noise covariance of z was obtained as follows [12,20]:

(4) $$\begin{array}{cc} R & =\left[\begin{array}{cc} R^{11} & R^{12}\\ R^{12} & R^{22} \end{array}\right],\\ R_{11} & =r_{m}^{2}\;\sigma_{\theta }^{2}\;sin^{2}\left(\theta_{m}\right)+\sigma_{r}^{2}\;cos^{2}\left(\theta_{m}\right),\\ R_{22} & =r_{m}^{2}\;\sigma_{\theta }^{2}\;cos^{2}\left(\theta_{m}\right)+\sigma_{r}^{2}\;sin^{2}\left(\theta_{m}\right),\\ R_{12} & =\left(\sigma_{r}^{2}-r_{m}^{2}\;\sigma_{\theta }^{2}\right)\;sin\left(\theta_{m}\right)\;cos\left(\theta_{m}\right). \end{array}$$

Equation (4) indicates that R depends on the measurements of $r_{m}$ and $\theta_{m}$. Thus, R varied with time, which is because $r_{m}$ and $\theta_{m}$ varied with time owing to random noises.

Typically, stochastic filters (state estimators) are used in radar-based target tracking to reduce the effects of the measurement noise. The stochastic filtering (state estimation) requires state-space system models that are composed of motion and measurement models. In the preceding vehicle tracking, the state of the target is defined as $x={\left[x\;v_{x}\;y\;v_{y}\right]}^{T}$, where $v_{x}$ and $v_{y}$ are the 2D velocities along the *x*- and *y*-axes. If the state vector at a discrete time step *k* is defined as $x\left(k\right)={\left[x\left(k\right)\;v_{x}\left(k\right)\;y\left(k\right)\;v_{y}\left(k\right)\right]}^{T}$, the motion model can be represented

(5) $$\begin{array}{cc} x(k+1) & =Fx(k)+Gw(k),\\ F & =\left[\begin{array}{cccc} 1 & T & 0 & 0\\ 0 & 1 & 0 & 0\\ 0 & 0 & 1 & T\\ 0 & 0 & 0 & 1 \end{array}\right],\;\;G=\left[\begin{array}{cc} T^{2}/2 & 0\\ T & 0\\ 0 & T^{2}/2\\ 0 & T \end{array}\right], \end{array}$$

where *T* is the sampling interval and w(k) is the white Gaussian process noise that represents the acceleration. Therefore, the motion model (5) is referred to as the discrete white noise acceleration (DWNA) model. The covariance of w(k) can be represented as $Q\left(k\right)=\sigma_{w}^{2}\;I_{2}$, where $I_{2}$ is the 2×2 identity matrix.

The measurement model expresses the relationship between measurement and state. If the measurement vector at time *k* is defined as $z\left(k\right)={\left[x_{m}\left(k\right)\;y_{m}\left(k\right)\right]}^{T}$, the measurement model can be represented as:

(6) $$\begin{array}{cc} z(k) & =Hx\left(k\right)+\bar{v}\left(k\right),\\ H & =\left[\begin{array}{cccc} 1 & 0 & 0 & 0\\ 0 & 0 & 1 & 0 \end{array}\right], \end{array}$$

where $\bar{v}\left(k\right)$ is the measurement noise vector, and its covariance is R(k). As previously mentioned, R(k) varies with time, and should be computed at each time step using (4).

PDAF is one of the most renowned stochastic filter for single target tracking in clutter. PDAF is the combination of the probabilistic data association (PDA) algorithm and the Kalman filter (KF). In this filter, PDA handles the clutter, and KF reduces the effects of measurement noises. KF recursively updates the state estimate and the estimation error covariance at each time step, indicating that it requires an initial state estimate and estimation error covariance. PDAF also requires the initial estimation information. If there is no clutter (i.e., clean environment) and only one measurement is obtained at each time step, the initial information can be easily obtained using a two-point differencing method as follows:

(7) $$\begin{array}{cc} \hat{x}\left(0\right) & =\left[\begin{array}{c} \hat{x}\left(0\right)\\ {\hat{v}}_{x}\left(0\right)\\ \hat{y}\left(0\right)\\ {\hat{v}}_{y}\left(0\right) \end{array}\right]=\left[\begin{array}{c} x_{m}\left(0\right)\\ (x_{m}\left(0\right)-x_{m}\left(-1\right))/T\\ y_{m}\left(0\right)\\ (y_{m}\left(0\right)-y_{m}\left(-1\right))/T \end{array}\right], \end{array}$$

(8) $$\begin{array}{cc} P_{k}\left(0\right) & =\left[\begin{array}{cccc} R_{11}\left(0\right) & R_{11}\left(0\right)/T & 0 & 0\\ R_{11}\left(0\right)/T & 2R_{11}\left(0\right)/T^{2} & 0 & 0\\ 0 & 0 & R_{22}\left(0\right) & R_{22}\left(0\right)/T\\ 0 & 0 & R_{22}\left(0\right)/T & 2R_{22}\left(0\right)/T^{2} \end{array}\right], \end{array}$$

where the time step, k=0, indicates that $\hat{x}\left(0\right)$ and $P_{k}\left(0\right)$ are the initial information and the actual estimation starts at k=1. In addition, k=−1 indicates that it is one time step earlier than k=0.

In real situations, false measurements (clutters) hinders the initialization process. Track formation algorithm determines the initial information required to start the actual tracking (which is called the track maintenance). Therefore, this study focused on the track formation for preceding vehicle tracking in highly heavy clutter environment, which has been a challenging problem. In the following sections, we introduce an existing track formation algorithm and the novel track formation algorithm proposed in this study.

## 3. Multiple Model Track Formation and Problem Formulation

In this section, we introduce a state-of-the-art track formation algorithm known as the MMTF. The MMTF begines with track initiation, which involves the generation of preliminary tracks via the combination of measurements at times k=1 and k=2. If the number of measurements obtained at times *k* is denoted as m(k), m(1)×m(2) pairs of measurements can be obtained. However, the number of measurement pairs in highly-heavy clutter environment is very large (more than a thousand). Among the large number of measurement pairs, unreasonable pairs that exceed a maximum possible velocity are discarded through the gating technique. The gate is defined as a rectangle with an area of

(9) $$\begin{array}{cc} V_{\mathrm{rect}} & =[2\left(v_{x,\max}\times T+2\sqrt{R_{11}}\right][2\left(v_{y,\max}\times T+2\sqrt{R_{22}}\right], \end{array}$$

where $v_{x,\max}$ and $v_{y,\max}$ are the maximum possible velocities along with the *x*- and *y*-axes, respectively. The measurement pairs remaining after the gating process are known as the preliminary tracks, where “track” indicates a sequence (history) of measurements or the estimation results (state estimate and covariance) obtained from the sequence of measurements. Subsequently, a two-point differencing is performed using the preliminary tracks to obtain the initial state estimates and their covariances. The second process, known as the preliminary tracking, is performed from k=3 to $k=N_{W}$, where $N_{W}$ is the window length of the MMTF. To this end, an interacting multiple model probability density filter (IMMPDAF) is used with the following motion models [12]:

- $M_{1}$: DWNA model (5) assuming undetected target (false target)
- $M_{2}$: DWNA model (5) assuming detected target (true target)

The only difference between $M_{1}$ and $M_{2}$ is the target detection probability, which can be expressed as follows:

(10) $$\begin{array}{cc} P_{D}^{j} & =\left\{\begin{array}{c} 0,\;\;\mathrm{if}\;\;j=1,\\ P_{D},\;\;\mathrm{if}\;\;j=2, \end{array}\right. \end{array}$$

where *j* is the model (mode) number. The processes of the IMMPDAF can be summarized as follows:

1. Compute the mixing probabilities:
  (11) $$\begin{array}{cc} \mu_{i|j}(k-1|k-1)= & \frac{1}{{\bar{c}}_{j}}p_{ij}\mu_{i}\left(k-1\right), \end{array}$$
  (12) $$\begin{array}{cc} {\bar{c}}_{j}= & \sum_{i=1}^{r}p_{ij}\mu_{i}\left(k-1\right). \end{array}$$
2. Obtain an initial estimate and a covariance for the model-matched filter, *j* (j=1,2):
  (13) $$\begin{array}{cc} {\hat{x}}^{0j}\left(k-1|k-1\right) & =\sum_{i=1}^{r}{\hat{x}}^{0j}\left(k-1|k-1\right)\mu_{i|j}\left(k-1|k-1\right), \end{array}$$
  (14) $$\begin{array}{cc} P^{0j}\left(k-1|k-1\right) & =\sum_{i=1}^{r}\mu_{i|j}\left(k-1|k-1\right)\{P^{i}\left(k-1|k-1\right)+[{\hat{x}}^{i}\left(k-1|k-1\right)\\ \; & -{\hat{x}}^{0j}\left(k-1|k-1\right)]{\left[{\hat{x}}^{i}\left(k-1|k-1\right)-{\hat{x}}^{0j}\left(k-1|k-1\right)\right]}^{T}\}. \end{array}$$
3. Perform a mode-conditioned filtering using PDAF (see Appendix A), and obtain ${\hat{x}}^{j}\left(k|k\right)$ and $P^{j}\left(k|k\right)$ for j=1,2.
4. Compute the likelihood functions:
  (15) $$\begin{array}{cc} \Lambda_{j}\left[Z\left(k\right)\right] & =\frac{1-P_{D}^{j}}{V^{m(k)}}+\frac{m\left(k\right)P_{D}^{j}}{V^{m(k)-1}}\sum_{i=1}^{m(k)}N\left[z_{i}\left(k\right);{\hat{z}}^{j}\left(k|k-1\right),S^{j}\left(k\right)\right], \end{array}$$
  (16) $$\begin{array}{cc} V & =c_{n_{z}}\gamma^{n_{z}/2}{\left|S^{j}\left(k\right)\right|}^{1/2}, \end{array}$$
  (17) $$\begin{array}{cc} S^{j}\left(k\right) & =HP^{j}\left(k|k\right)H^{T}+R\left(k\right), \end{array}$$
  (18) $$\begin{array}{cc} {\hat{z}}^{j}\left(k|k-1\right) & =H{\hat{x}}^{j}\left(k|k\right), \end{array}$$
  where $N[z_{i}\left(k\right);{\hat{z}}^{j}\left(k|k-1\right),S^{j}\left(k\right)]$ indicates the Gaussian probability density function with the mean ${\hat{z}}^{j}\left(k|k-1\right)$ and covariance $S^{j}\left(k\right)$; Z(k) is the set of validated measurements defined as $Z\left(k\right)={\left\{z_{i}\left(k\right)\right\}}_{i=1}^{m(k)}$; and $z_{i}\left(k\right)$ is the *i*-th measurement obtained at time *k*.
5. Update the mode probabilities:
  (19) $$\begin{array}{cc} \mu_{j}\left(k\right) & =\frac{1}{c}\Lambda_{j}\left[Z\left(k\right)\right]{\bar{c}}_{j}, \end{array}$$
  (20) $$\begin{array}{cc} c & =\sum_{j=1}^{r}\Lambda_{j}\left[Z\left(k\right)\right]{\bar{c}}_{j}. \end{array}$$
6. Obtain the estimate and covariance at time *k* by combination using:
  (21) $$\begin{array}{cc} \hat{x}\left(k|k\right) & =\sum_{j=1}^{r}{\hat{x}}^{j}\left(k|k\right)\mu_{j}\left(k\right), \end{array}$$
  (22) $$\begin{array}{cc} P(k|k) & =\sum_{j=1}^{r}\mu_{j}\left(k\right)\{P^{j}\left(k|k\right)+\left[{\hat{x}}^{j}\left(k|k\right)-\hat{x}\left(k|k\right)\right]\\ \; & \times {\left[{\hat{x}}^{j}\left(k|k\right)-\hat{x}\left(k|k\right)\right]}^{T}\}, \end{array}$$
  where $\hat{x}\left(k|k\right)$ and P(k|k) are the a posteriori (i.e., the measurements up to time *k* were used for estimation at time *k*) estimated state and estimation error covariance. In addition, $\hat{x}\left(k|k-1\right)$ and P(k|k−1) indicate a priori (i.e., the measurements up to time k−1 were used for estimation at time *k*) estimated state and covariance.

At the initial time of preliminary tracking, k=3, the mode probabilities were equally set as $\mu_{j}\left(3\right)=0.5$. The transition probabilities from mode *i* to *j*, denoted as $p_{ij}$, were defined by the transition probability matrix as:

(23) $$\begin{array}{c} \begin{array}{ccc} & M_{1} & M_{2}\\ M_{1} & 0.98 & 0.02\\ M_{2} & 0.02 & 0.98 \end{array} \end{array}$$

The mode probability of $M_{2}$, denoted as $\mu_{2}\left(k\right)$, corresponds to the probability that a track belongs to a true target. Thus, $\mu_{2}\left(k\right)$ is referred to as the true target probability (TTP). During the preliminary tracking, tracks with extremely low TTP (e.g., smaller than 0.05) are discarded. This pruning results in a gradual decrease in the number of tentative tracks. In the preceding vehicle tracking where there is only one target vehicle, there should be only one track at the final time of preliminary tracking, $k=N_{W}$. The track with the highest TTP is selected as the most reliable track.

As previously described, the MMTF is based on the two-point differencing method that is simple, but not accurate. Consequently, this significantly reduces the accuracy of the entire MMTF algorithm. Moreover, the TTP-based pruning cannot effectively reduce the number of tentative tracks in highly heavy clutter environments, which results in the computational burdening of the tracking algorithm.

## 4. FIR Filter-Based Track Formation

In this section, the novel track formation algorithm (i.e., the FFTF) proposed in this study is introduced. The most significant difference between the FFTF and the MMTF is in the track initiation process. Recursive filters including PDAF require an initial estimate and a covariance, which can be obtained using initialization methods (e.g., two-point differencing). However, FIR filters do not require the initialization, and can generate state estimate and covariances using recent finite number of measurements. Thus, FIR filters can be used for track initiation. Among various FIR filters, the minimum variance FIR filter (MVFF) was adopted in this study because it can produce not only the state estimate but also an estimation error covariance. The time-varying MVFF was proposed in [24] and was applied to localization problems in [21,22,23]. The equations of MVFF are written as follows:

(24) $$\begin{array}{cc} \hat{x}\left(k\right) & =L\;Z_{N}, \end{array}$$

(25) $$\begin{array}{cc} P(k) & =K_{N}Q_{N}K_{N}^{T}+LR_{N}L^{T}, \end{array}$$

(26) $$\begin{array}{cc} L & =J_{N}{\left[\begin{array}{cc} M_{1,1} & M_{1,2}\\ M_{1,2}^{T} & M_{2,2} \end{array}\right]}^{-1}\left[\begin{array}{c} {\bar{C}}_{N}^{T}\\ {\bar{G}}_{N}^{T} \end{array}\right]R_{N}^{-1}, \end{array}$$

(27) $$\begin{array}{cc} J_{N} & =[F^{N}\;\;F^{N-1}\;\;F^{N-2}\;\;\cdots \;\;F\;\;I], \end{array}$$

(28) $$\begin{array}{cc} M_{1,1} & ={\bar{C}}_{N}^{T}R_{N}^{-1}{\bar{C}}_{N},\\ M_{1,2} & ={\bar{C}}_{N}^{T}R_{N}^{-1}{\bar{G}}_{N},\\ M_{2,2} & ={\bar{G}}_{N}^{T}R_{N}^{-1}{\bar{G}}_{N}+Q_{N}^{-1}, \end{array}$$

(29) $$\begin{array}{cc} {\bar{C}}_{N} & =\left[\begin{array}{c} H\\ HF\\ HF^{2}\\ \vdots \\ HF^{N-1} \end{array}\right], \end{array}$$

(30) $$\begin{array}{cc} {\bar{G}}_{N} & =\left[\begin{array}{ccccc} 0 & 0 & \cdots & 0 & 0\\ HG & 0 & \cdots & 0 & 0\\ HFG & HG & \cdots & 0 & 0\\ \vdots & \vdots & \vdots & \vdots & \vdots \\ HF^{n-1}G & HF^{n-2}G & \cdots & HG & 0 \end{array}\right], \end{array}$$

(31) $$\begin{array}{cc} R_{N} & =\left[\mathrm{diag}(\overset{N}{\overset{︷}{R(k-N)\;R(k-N+1)\;\cdots \;R(k-1)}})\right], \end{array}$$

(32) $$\begin{array}{cc} Q_{N} & =\left[\mathrm{diag}(\overset{N}{\overset{︷}{Q(k-N)\;Q(k-N+1)\;\cdots \;Q(k-1)}})\right], \end{array}$$

(33) $$\begin{array}{cc} Z_{N} & ={\left[z^{T}\left(k-N\right)\;\;z^{T}\left(k-N+1\right)\;\;\cdots \;\;z^{T}\left(k-1\right)\right]}^{T}, \end{array}$$

(34) $$\begin{array}{cc} K_{N} & =[F^{N-1}G\;\;F^{N-2}G\;\;\cdots \;\;\mathrm{FG}\;\;G], \end{array}$$

where *N* is the horizon size of the MVFF, and *N* should be greater than or equal to the dimension of the state vector. As reported in (24)–(34), the MVFF produces state estimate and covariance at time *k* using the finite number of measurements on the horizon [k−N,k−1]. Considering the dimension of the state vector in (5), the MVFF requires a minimum of four measurements.

Preliminary tracks for the FFTF are obtained in a similar manner to that of the MMTF. The only difference is that measurements from k=1 to k=4 are used for the FFTF. As shown in (24), the MVFF produces a state estimate by multiplying the filter gain L and the augmented measurement vector $Z_{N}$. L can be obtained using (26)–(31), where F and G are defined in (5); H is defined in (6); and Q(k) and R(k) are process and measurement noise covariances, respectively. In addition, $Z_{N}$ can be obtained using (33). Combining measurements from k=1 to k=4, a large number of $Z_{N}$ can be obtained. The number of combinations for making $Z_{N}$ is $\prod_{k=1}^{4}m\left(k\right)$, which is the same as preliminary tracks produced by the MVFF. This method generates a significantly higher number of preliminary tracks than the MMTF. To effectively reduce the number of tentative tracks, we proposed a drastic method that selects one track for each measurements at a certain time. For a measurement $z_{i}\left(k\right)$, the most reliable track was selected by evaluation based on the Mahalanobis distance [21,22,25] defined as follows:

(35) $$\begin{array}{cc} D_{i,h}\left(k\right) & ={\left[z_{i}\left(k\right)-{\hat{z}}_{h}\left(k\right)\right]}^{T}R_{i}^{-1}\left(k\right)\left[z_{i}\left(k\right)-{\hat{z}}_{h}\left(k\right)\right], \end{array}$$

(36) $$\begin{array}{cc} {\hat{z}}_{h}\left(k\right) & =H{\hat{x}}_{h}\left(k\right), \end{array}$$

where $D_{i,h}\left(k\right)$ is the Mahalanobis distance [25] between the actual measurement $z_{i}\left(k\right)$ and the predicted measurement ${\hat{z}}_{h}\left(k\right)$. ${\hat{x}}_{h}\left(k\right)$ is the estimate obtained from the *h*-th tentative track, where h=1,2,⋯,n(k), and n(k) is the number of tentative tracks at time *k*. The estimate and covariance at time k=5 are obtained using the MVFF and the combination of measurements (preliminary tracks) from k=1 to k=4, and those of the times from k=6 to $k=N_{W}$ are obtained using the PDAF (see Appendix A). The estimate ${\hat{x}}_{h}\left(k\right)$ for h=1,2,⋯,n(k) produced the minimum $D_{i,h}\left(k\right)$ was selected as the most reliable track for $z_{i}\left(k\right)$.

There are some similarities between the proposed track selection method and the resampling process in particle filtering. In the resampling process, a certain number of samples (i.e., state estimates) are selected based on the likelihood. In this process, a sample with a high likelihood may be repeatedly selected. Consequently, the number of samples with high likelihoods increases over time, which is an example of the survival of the fittest. The track in our method corresponds to the sample in particle filtering. Tracks are selected at each times step in the preliminary tracking (from k=6 to $k=N_{W}$), in which the Mahalanobis distance is used as an evaluation tool. Each measurement has the authority to select a track that has the shortest Mahalanobis distance. A track may be selected for two or more measurements. Consequently, more favored (reliable) tracks can generate their duplicates, which can be viewed as the survival of the fittest.

At the initial time of preliminary tracking, the number of tentative tracks, n(k), is significantly higher than the number of measurements, m(k), in highly-heavy clutter environments. However, at k=5, n(k) drastically decreased to m(k) by the selection based on the Mahalanobis distance. Moreover, n(k) maintained the same value as m(k) during the preliminary tracking. At the final time of the preliminary tracking, the tentative tracks were merged by averaging to obtain the ultimate estimate and covariance. The overall algorithm of the FFTF is summarized in Algorithm 1, where the a posteriori estimate and covariance were simply denoted as $\hat{x}\left(k\right)$ and P(k), respectively.

**Algorithm 1:** FFTF

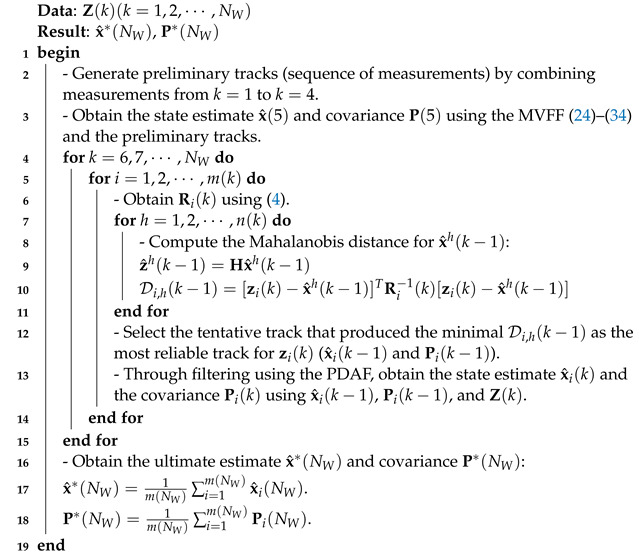



## 5. Simulation Results

In this section, the simulation results of preceding vehicle tracking using an automotive radar are presented to demonstrate the performance of the proposed FFTF. For the simulation, a typical situation of preceding vehicle tracking using the adaptive cruise control system on the highway was considered. The simulation scenario is as follows. The preceding and host vehicles are in the same lane. Various conditions for the relative distance, $D_{r}$, and velocity, $V_{r}$, between them were considered. In addition, the automotive radar was assumed to be the Delphi Electronically Scanning Radar (ESR), which has two operation modes. In the long-range mode, the radar exhibited an FOV and maximum detection range of $20^{\circ }$ and 174 m, respectively. In the mid-range mode, the radar exhibited an FOV and maximum detection range of $90^{\circ }$ and 60 m, respectively. Next, highly-heavy clutter environments were assumed, and the clutter density was set as $\lambda =1.0\times 10^{-1}$. This clutter density enabled the generation of more than 30 false measurements (clutters) at each time step. False measurements were generated so that they are independently and uniformly distributed in a square centered at the correct measurement. The area of the square *V* was computed as follows:

(37) $$\begin{array}{cc} V & =N_{F}/\lambda , \end{array}$$

(38) $$\begin{array}{cc} N_{F} & ={\left[10V\left(k\right)\lambda +1\right]}^{-}, \end{array}$$

(39) $$\begin{array}{cc} V(k) & ={\pi \gamma |S\left(k\right)|}^{1/2}, \end{array}$$

where $N_{F}$ is the number of false measurements; the notation ${\left[\cdot \right]}^{-}$ indicates “rounded down to the nearest integer”; and S(k) was obtained using KF under the assumption of an ideal clean (no clutters) environment [12].

The sampling interval was set as T=0.1s. The target detection probability was set as $P_{D}=0.9$. The automotive radar reported range/bearing measurements with noise covariances, $\sigma_{r}=0.25$ m and $\sigma_{theta}=1.5{\;}^{\circ }$ [26,27]. The gate probability and the gate threshold were set as $P_{G}=0.99$ and γ=9.21, respectively. Trajectories of preceding vehicle were generated using the DWNA model (5) with $\sigma_{w}=0.08$.

Using the aforementioned scenario and parameters, the preceding vehicle tracking was simulated only up to the track formation stage ($1\leq k\leq N_{W}$). The track formation was conducted using MMTF and FFTF, and their accuracies were compared in terms of the root-mean-square position error (RMSPE) and the root-mean-square velocity error (RMSVE) at the final time of track formation, $k=N_{W}$. The RMSPE and RMSVE are defined as follows:

(40) $$\begin{array}{cc} \mathrm{RMSPE}(k) & =\frac{1}{N_{MC}}\sum_{i=1}^{N_{MC}}\sqrt{{\left(x\left(k\right)-{\hat{x}}^{i}\left(k\right)\right)}^{2}+{\left(y\left(k\right)-{\hat{y}}^{i}\left(k\right)\right)}^{2}}, \end{array}$$

(41) $$\begin{array}{cc} \mathrm{RMSVE}(k) & =\frac{1}{N_{MC}}\sum_{i=1}^{N_{MC}}\sqrt{{\left(v_{x}\left(k\right)-{\hat{v}}_{x}^{i}\left(k\right)\right)}^{2}+{\left(v_{y}\left(k\right)-{\hat{v}}_{y}^{i}\left(k\right)\right)}^{2}}, \end{array}$$

where $N_{MC}$ is the number of Monte Carlo (MC) runs; (x(k),y(k)) and $\left(v_{x}\left(k\right),v_{y}\left(k\right)\right)$ are the true 2D positions and velocities, respectively; and $\left({\dot{x}}^{i}\left(k\right),{\dot{y}}^{i}\left(k\right)\right)$ and $\left({\hat{v}}_{x}^{i}\left(k\right),{\hat{v}}_{y}^{i}\left(k\right)\right)$ are the estimated 2D positions and velocities obtained the in *i*-th MC run, respectively.

For the window length of track formation, $N_{W}$, various values from 6 to 20 were tested. For each value of $N_{W}$, 100 MC simulations were performed, and the RMSPE and RMSVE at the final time of track formation were computed. The relative distance and velocity between the preceding and host vehicles were denoted as $D_{r}$ and $V_{r}$.

**Figure 2:**
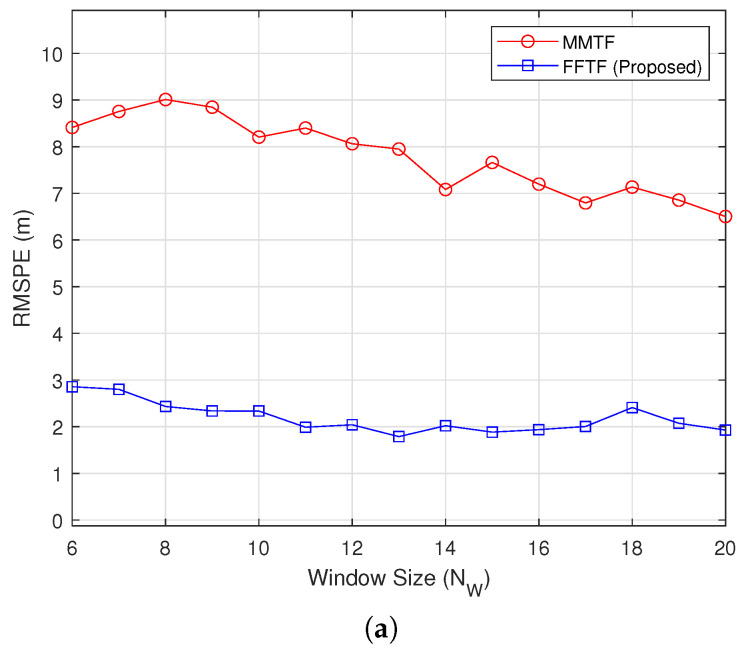

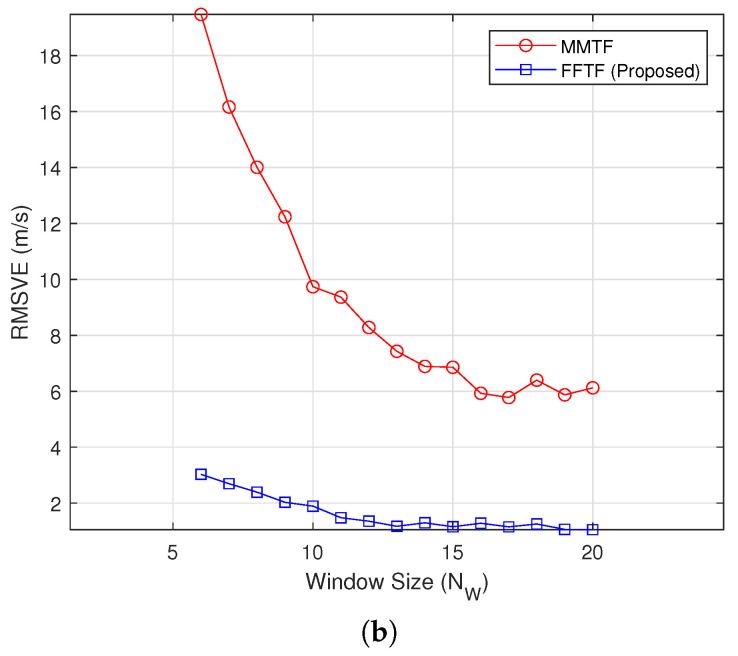
Simulation results under the long-range mode ($D_{r}=100$ m): (**a**) root-mean-square position error (RMSPE), (**b**) root-mean-square velocity error (RMSVE).

Figure 2 shows the simulation results under the long-range mode at a relative distance of $D_{r}=100$ m, during which the distance was maintained ($V_{r}=0$ km/h). The proposed FFTF exhibited significantly smaller RMSPE and RMSVE values than the MMTF algorithm. Moreover, the FFTF exhibited a consistent performance, whereas the RMSVE values of the MMTF decreased with a decrease in $N_{W}$.

**Figure 3:**
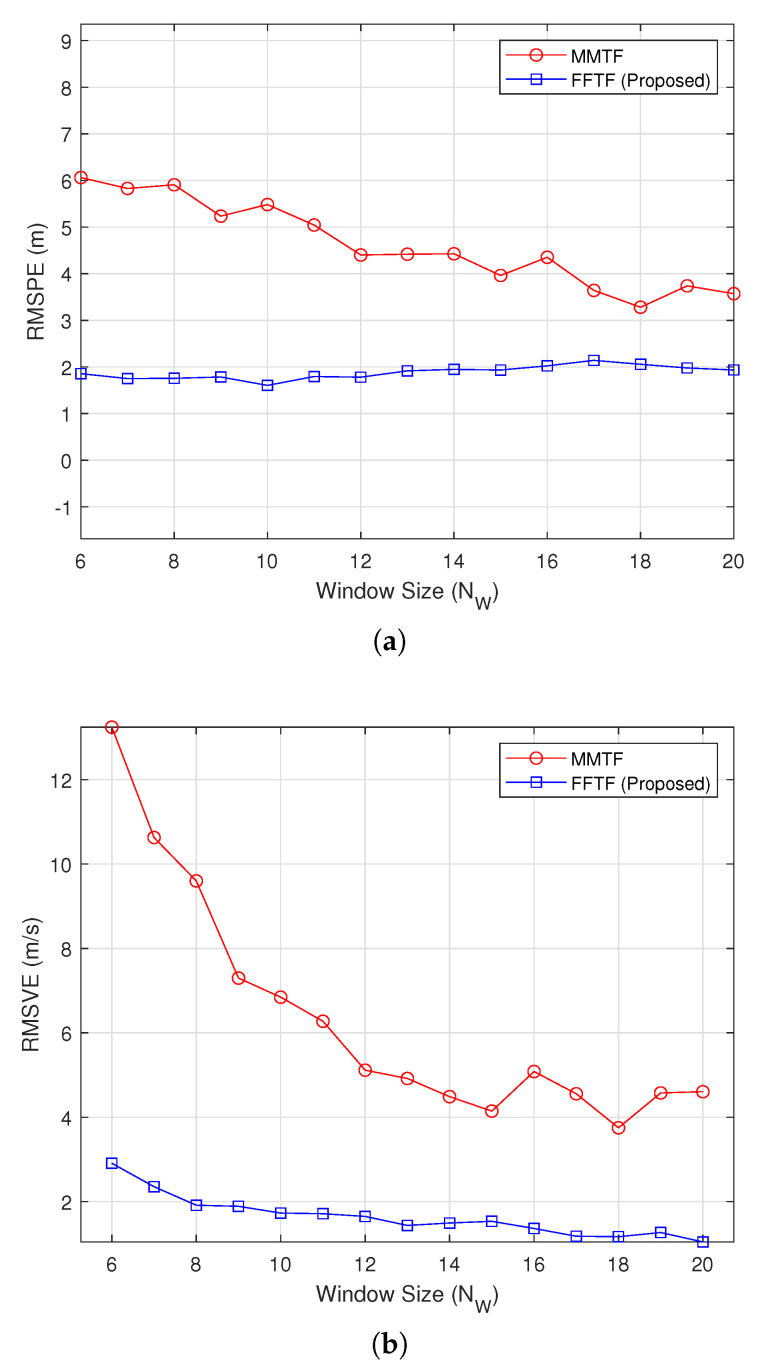
Simulation results under the mid-range mode ($D_{r}=50$ m): (**a**) RMSPE; (**b**) RMSVE.

Figure 3 shows the simulation results under the mid-range mode at a relative distance of $D_{r}=50$ m, during which the distance was maintained ($V_{r}=0$ km/h). The proposed FFTF outperformed the MMTF under this condition, but the performance gap was smaller than that of the long-range condition. The largest performance gap was observed at $N_{W}=6$.

**Figure 4:**
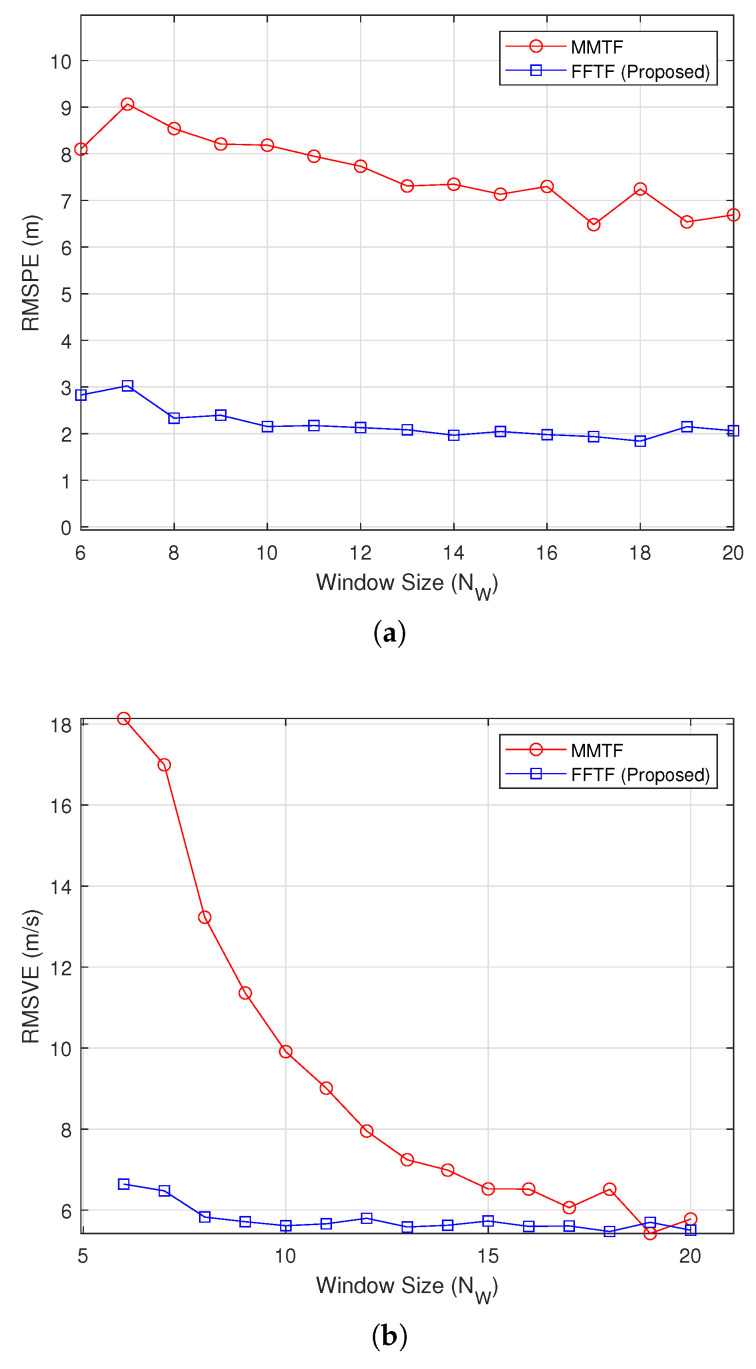
Simulation results under the getting closer scenario ($V_{r}=-20$ km/h): (**a**) RMSPE; (**b**) RMSVE.

In this study, different scenarios, in which the relative distance changed due to the different velocities of the two vehicles, were considered. The velocity of the preceding and host vehicles along the road were denoted as $V_{p}$ and $V_{h}$, respectively. Accordingly, the relative velocity was defined as $V_{r}=V_{p}-V_{h}$. Initially, $V_{r}=-20$ km/h was used, during which the two vehicles moved closer to each other. The initial distance between the vehicles was set to 100 m. Figure 4 shows the simulation results obtained under this scenario. The FFTF outperformed the MMTF, and the performance gap between both algorithms in terms of the RMSVE decreased as $N_{W}$ increased. In addition, as the window size of the track formation algorithm increases, the time consumption before the actual tracking began increased. This indicates that a large $N_{W}$ is not suitable.

**Figure 5:**
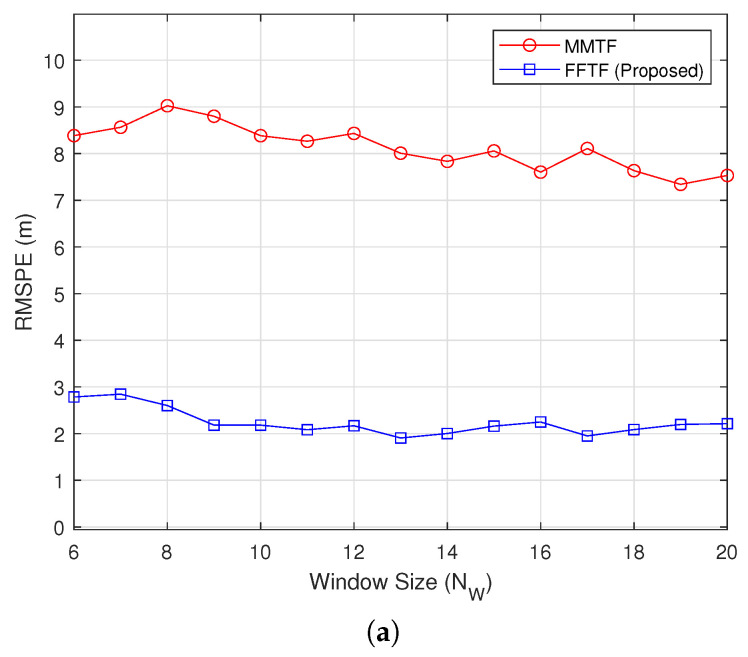

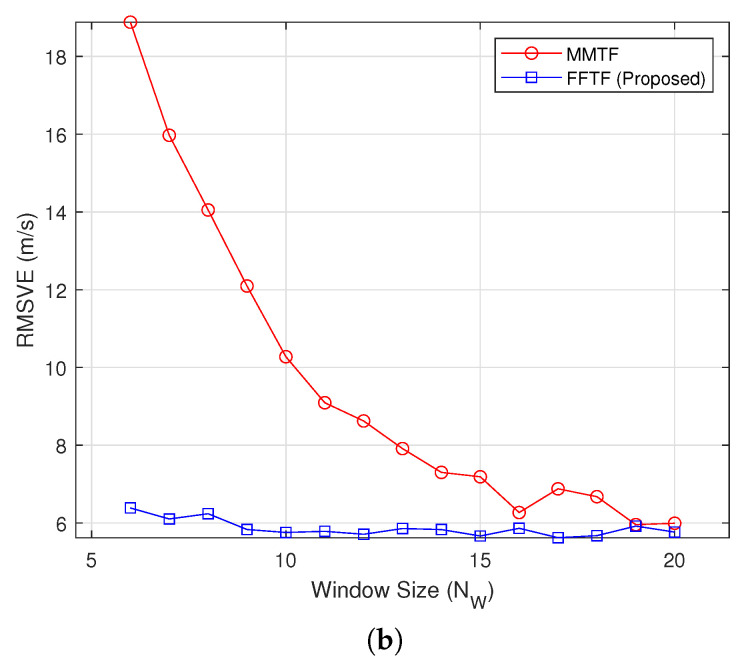
Simulation results under getting farther scenario ($V_{r}=+20$ km/h): (**a**) RMSPE; (**b**) RMSVE.

Next, simulation was conducted under a scenario where the vehicles were getting farther (Figure 5). Here, $V_{r}$ was set to 20 km/h, and the initial $D_{r}$ was set to 100 m. The simulation results obtained under the “getting farther” scenario were similar to obtained under the “getting closer” scenario. In addition, FFTF outperformed MMFT, but the performance gap in terms of the RMSVE value decreased as $N_{W}$ increased. Because the sampling interval was T=0.1 s, the time consumption for the track formation at $N_{W}=20$ was 2 s. On the highway, a fast reaction is required, indicating that a time delay of 2 s is not suitable. In this study, using the FFTF at $N_{W}=6$, both a fast reaction with a short time delay and accurate track formation were achieved.

**Figure 6:**
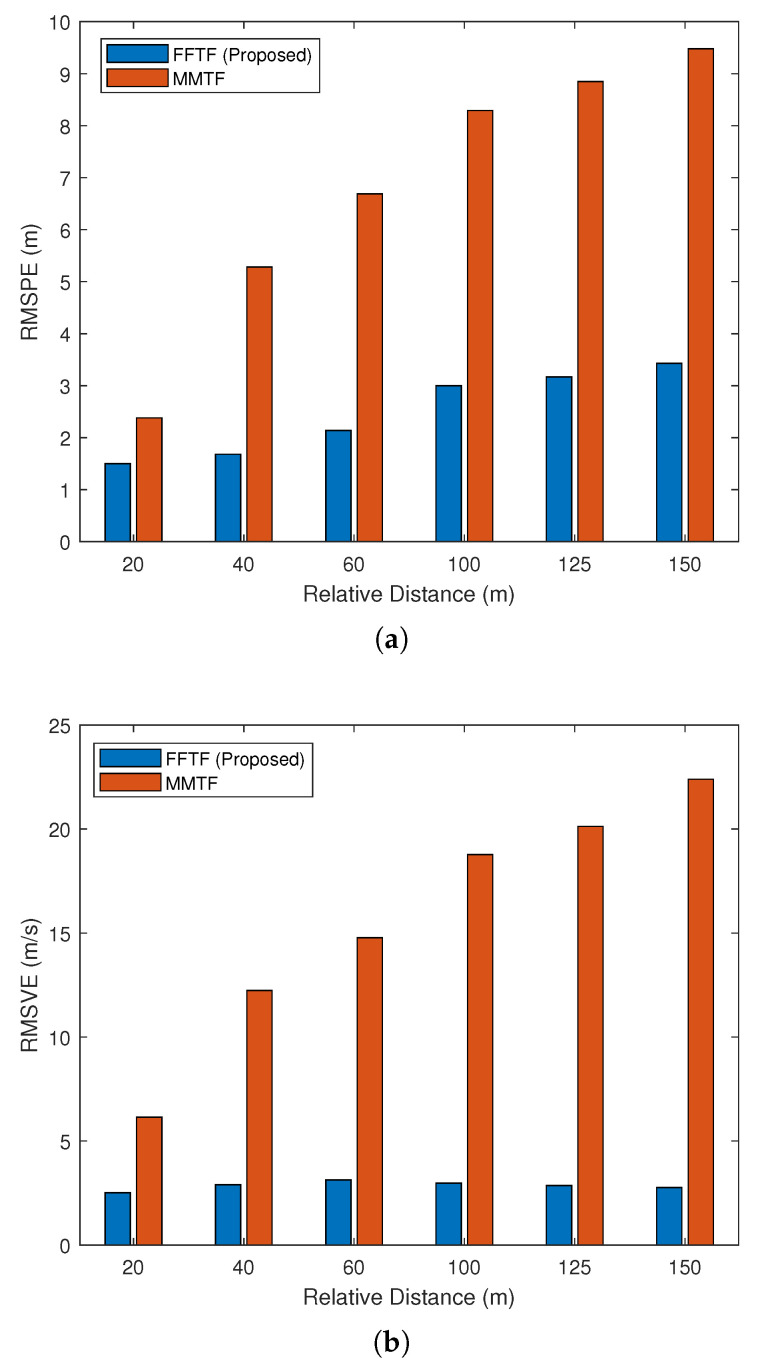
Simulation results under various relative distances: (**a**) RMSPE; (**b**) RMSVE.

Therefore, simulations were performed under more diverse $D_{r}$ and $V_{r}$ conditions at $N_{W}=6$. Table 1 and Table 2 show the simulation results obtained under various relative distances, which are represented more intuitively in Figure 6. Table 3 shows the results obtained with various relative velocities. The RMSPE and RMSVE values increased as $D_{r}$ and $V_{r}$ increased. Compared to the MMTF algorithm, the proposed FFTF algorithm exhibited not only a higher accuracy but also more consistent performance despite the changes in the $D_{r}$ and $V_{r}$ conditions.

## 6. Conclusions

This study proposed a novel track formation algorithm, namely FFTF, for preceding vehicle tracking using automotive radars. In the proposed FFTF algorithm, first, the initial estimate and covariance were obtained using a type of FIR filter (i.e., MVFF). The FFTF effectively reduced the number of preliminary/tentative tracks using the Mahalanobis distance-based evaluation. In the simulations, the FFTF was significantly more accurate than the MMTF in terms of both RMSPE and RMSVE. The FFTF exhibited consistent performance, whereas the MMTF exhibited large changes in performance depending on the window size, relative distance and velocities. Therefore, the FFTF algorithm is expected to exhibit improved performance for preceding vehicle tracking in automotive radar systems. However, the FFTF requires more computation time for track initiation process compared to the MMTF. We intend to improve the FFTF by adopting a new FIR filter in future work.

## Figures and Tables

**Table 1:** Simulation results under various relative distances in the long-range mode.

Performance	100 m		125 m		150 m	
Metric	MMTF	FFTF	MMTF	FFTF	MMTF	FFTF
RMSPE (m)	8.29	3.00	8.85	3.17	9.48	3.43
RMSVE (m/s)	18.77	2.98	20.13	2.86	22.39	2.76

**Table 2:** Simulation results under various relative distances in the mid-range mode.

Performance	20 m		40 m		60 m	
Metric	MMTF	FFTF	MMTF	FFTF	MMTF	FFTF
RMSPE (m)	2.38	1.50	5.28	1.68	6.69	2.14
RMSVE (m/s)	6.15	2.51	12.24	2.90	14.77	3.13

**Table 3:** Simulation results under various relative velocities in the long-range mode.

Performance	−30 km/h		−10 km/h		+10 km/h		+30 km/h	
Metric	MMTF	FFTF	MMTF	FFTF	MMTF	FFTF	MMTF	FFTF
RMSPE (m)	8.10	3.09	7.85	3.04	8.55	3.15	8.65	3.01
RMSVE (m/s)	18.41	9.02	17.86	4.53	18.90	4.16	19.79	9.06

## Abbreviations

The following abbreviations are used in this manuscript:

FIR	Finite Impulse Response
PDAF	Probabilistic Data Association Filter
IMM	Interacting Multiple Model
MMTF	Multiple Model Track Formation
FFTF	FIR Filter-Based Track Formation
IMMPDAF	Interacting Multiple Model Probabilistic Data Association Filter
2D	Two-Dimensional
KF	Kalman Filter
DWNA	Discrete White Noise Acceleration
TTP	True Target Probability
PDA	Probabilistic Data Association
MVFF	Minimum Variance FIR Filter
RMSPE	Root-Mean-Square Position Error
RMSVE	Root-Mean-Square Velocity Error

## Appendix A. Probabilistic Data Association Filter

The probabilistic data association filter (PDAF) algorithm commonly used in the MMTF and FFTF is introduced in this appendix. The PDAF recursively performed the following processes. The state estimate and covariance were updated using the motion model:

(A1) $$\begin{array}{cc} \hat{x}\left(k|k-1\right) & =F\hat{x}\left(k-1|k-1\right),\\ P(k|k-1) & =FP\left(k-1|k-1\right)F^{T}+GQ\left(k\right)G^{T}. \end{array}$$

The measurements within the validation region (i=1,⋯,m(k)) are selected:

(A2) $$\begin{array}{cc} \nu_{i}\left(k\right) & ={\left(z_{i}\left(k\right)-\hat{z}\left(k\right)\right)}^{T}S_{i}^{-1}\left(k\right)\left(z_{i}\left(k\right)-\hat{z}\left(k\right)\right)\leq \gamma ,\\ S_{i}\left(k\right) & =HP\left(k|k-1\right)H^{T}+R_{i}\left(k\right), \end{array}$$

where $\nu_{i}\left(k\right)$ is the innovation for $z_{i}\left(k\right)$; γ is the gate threshold; and $S_{i}\left(k\right)$ is the innovation covariance. The gate threshold γ can be obtained from a *chi-square* table.

The association probability for $z_{i}\left(k\right)$ being the correct measurement is computed:

(A3) $$\begin{array}{cc} \beta_{i}\left(k\right) & =\left\{\begin{array}{c} \frac{L_{i}\left(k\right)}{1-P_{D}P_{G}+\sum_{j=1}^{m(k)}L_{j}\left(k\right)}\;\;\;\;i=1,\cdots ,m\left(k\right)\\ \frac{1-P_{D}P_{G}}{1-P_{D}P_{G}+\sum_{j=1}^{m(k)}L_{j}\left(k\right)}\;\;\;\;i=0 \end{array}\right. \end{array}$$

where i=0 corresponds to “none is correct”, and m(k) is the number of measurements [12,28]. The likelihood ratio $L_{j}\left(k\right)$ is computed:

(A4) $$\begin{array}{cc} L_{j}\left(k\right) & =\frac{\exp[-0.5{\left(z_{j}\left(k\right)-\hat{z}\left(k\right)\right)}^{T}S_{j}^{-1}\left(k\right)\left(z_{j}\left(k\right)-\hat{z}\left(k\right)\right)]}{\left(2\pi \right)|R_{j}{\left(k\right)|}^{1/2}\;m\left(k\right)}\;P_{D}\;V_{j}\left(k\right)\\ V_{j}\left(k\right) & =c_{n_{z}}\gamma {\left|S_{j}\left(k\right)\right|}^{1/2}, \end{array}$$

where $V_{j}\left(k\right)$ is the volume of the validation region. The state estimate is updated using the measurements:

(A5) $$\begin{array}{cc} \hat{x}\left(k|k\right) & =\hat{x}\left(k|k-1\right)+\sum_{i=1}^{m(k)}W_{i}\left(k\right)\;\beta_{i}\left(k\right)\;\nu_{i}\left(k\right),\\ W_{i}\left(k\right) & =P\left(k|k-1\right)H^{T}S_{i}^{-1}\left(k\right),\\ \nu_{i}\left(k\right) & =z_{i}\left(k\right)-\hat{z}\left(k\right). \end{array}$$

In addition, the estimation error covariance was updated as follows [28]:

(A6) $$\begin{array}{cc} P(k|k) & =\bar{P}\left(k|k\right)+\tilde{P}\left(k|k\right),\\ \bar{P}\left(k|k\right) & =\beta_{0}\left(k\right)\;P\left(k|k-1\right)+\sum_{i=1}^{m(k)}\beta_{i}\left(k\right)\left(I-W_{i}\left(k\right)H\right)P\left(k|k-1\right),\\ \tilde{P}\left(k|k\right) & =\sum_{i=1}^{m(k)}\beta_{i}\left(k\right)W_{i}\left(k\right)\nu_{i}\left(k\right)\nu_{i}^{T}\left(k\right)W_{i}^{T}\left(k\right)\\ \; & -\left(\sum_{i=1}^{m(k)}\beta_{i}\left(k\right)W_{i}\left(k\right)\nu_{i}\left(k\right)\right)\left(\sum_{i=1}^{m(k)}\beta_{i}\left(k\right)\nu_{i}^{T}\left(k\right)W_{i}^{T}\left(k\right)\right). \end{array}$$

If there is no measurement in the validation region, the PDAF does not perform the measurement update processes.
